# Ginseng berry extract increases nitric oxide level in vascular endothelial cells and improves cGMP expression and blood circulation in muscle cells

**DOI:** 10.20463/jenb.2018.0018

**Published:** 2018-09-30

**Authors:** In-Ho Cho, Byung-Woo Kang, Park Yun-Jae, Han-Joo Lee, Sok Park, Namj Lee

**Affiliations:** 1 Human Performance Laboratory, Korea National Sport University, Seoul Republic of Korea; 2 Aribio central Research Institute, Pyeongtaek Republic of Korea; 3 Department of Physical Education, Sungkyul University, Anyang Republic of Korea; 4 Department of Physical Education, Kwangwoon University, Seoul Republic of Korea; 5 Department of Leisure Sports, Jungwon University, Goesan Republic of Korea

**Keywords:** ginseng berry extract, nitric oxide, cGMP expression, blood circulation

## Abstract

**[Purpose]:**

The purpose of this study was to determine whether ginseng berry extract improves blood circulation by regulating vasodilator expression in exposed to tumor necrosis factor alpha (TNF-α)-exposed endothelial cells and muscle cells.

**[Methods]:**

Nitric oxide (NO) and cGMP levels in human umbilical vein endothelial cells (HUVECs) and A7r5 cells exposed to ginseng berry extract were investigated. Based on the in vitro results, healthy participants were treated with ginseng berry extract for 4 weeks and then a non-invasive vascular screening device was used to confirm the improvement of blood circulation.

**[Results]:**

When TNF-α-treated cells were exposed to the ginseng berry extract, the expression levels of endothelial nitric oxide synthase (eNOS), NO, and cGMP were recovered to almost normal levels. In addition, TNF-ɑ-induced overexpression of vascular cell adhesion molecule 1 (VCAM-1), intracellular adhesion molecule 1 (ICAM-1), e-selectin, and p-selectin was lowered by ginseng berry extract. The ginseng berry extract significantly increased ankle brachial pressure index compared to placebo (p < 0.05).

**[Conclusion]:**

This study confirmed that the intake of ginseng berry extract improved blood circulation and therefore, its intake would be helpful for people having problems with blood circulation.

## INTRODUCTION

Blood via blood vessels delivers oxygen and nutrients to each tissue in the body and removes the waste produced by cells^1,2^, transports hormones, defends cells from external harmful substances, and maintains proper body temperature and homeostasis^3^. Therefore, a normal blood circulation is very important for maintenance of body function^4^.

The vascular endothelium is a layer of endothelial cells on the inner surface of the vascular layer^5^. It regulates fluidity and flow of blood and blood vessel tension via the synthesis and secretion of various kinds of substances having biological activity^6^. It also plays an important role in maintaining and controlling arterial blood vessel function and health by participating in platelet aggregation, thrombogenesis, and dissolution^7^. In other words, vascular endothelial cells primarily serve as an anatomical barrier to prevent blood circulation in the vessel wall, as well as to control blood vessel tension, partially control cell growth in the blood vessel wall, and regulate extracellular matrix deposition^8^. These cells are also involved in blood vessel protection from substances and cells, blood clotting in response to partial injury and inflammation, and homeostasis of blood vessels by repair responses^9^.

Continued inflammatory stimuli (e.g., oxidative stress, inflammatory cytokines, exposure to pathogenic agents) cause endothelial dysfunction, with endothelial cells becoming overactive or chronically active^10^. In this condition, damage to the vascular endothelium leads to excessive inflammation, resulting in excessive secretion of soluble adhesion molecules such as intercellular adhesion molecule (ICAM)-1, vascular cell adhesion molecule (VCAM)-1, e-selectin, and p-selectin from endothelial cells^11^. Oxidized cholesterol accumulates in the injured area, forming a plaque, and the inner wall of the blood vessel swells up, resulting in narrowing of the blood vessels and decreased blood circulation^11^.

Nitric oxide (NO), which plays a key role in maintaining endothelial homeostasis, is produced by endothelial nitric oxide synthase (eNOS) from the amino acid L-arginine in endothelial cells^12^. A typical function of NO is vasodilation^13^. The NO produced diffuses into the smooth muscle cells of the blood vessels and activates the soluble guanylate cyclase to induce cyclic guanosine-5'-monophosphate (cGMP)-mediated vascular relaxation^14^. cGMP acts as a second messenger to induce many biological effects of NO, such as relaxation of smooth muscle or inhibition of platelet aggregation^15^. NO inhibits the expression of proinflammatory cytokines, chemokines, and adhesion molecules in addition to vascular relaxation^16^. Therefore, it plays a key role in regulating vascular endothelial function, inhibiting vascular recruitment of white blood cells, proliferation of vascular smooth muscle cells, platelet aggregation, and inhibiting the production of tissue factor involved in thrombus formation^17^.

However, when functional and structural changes of vascular endothelial cells occur due to risk factors for vascular diseases such as hypertension, diabetes, hyperlipidemia, and smoking, endothelial cells cannot play the protective role mentioned above, but cause atherosclerosis^18^. NO production cannot be achieved by eNOS; NO is oxidized by reactive oxygen species to ONOO-, thus reducing the bioavailability of NO^19^. The resulting ONOO-oxidizes BH4, the coenzyme of eNOS, or increases the activity of arginase^20^. Therefore, eNOS uncoupling phenomenon (excessive O2 · - and H_2_O_2_ formation due to abnormal action of eNOS) is induced by decreasing L-arginine or insufficient BH4, which is the substrate of eNOS^21^. As a result, NO-mediated endothelium-dependent vascular dilation leads to impairment and negative effects on blood flow^22^.

Ginseng and its associated ingredients have been used in food or herbal medicines for thousands of years to treat many diseases^23^. It exhibits anti-aging, anti-diabetic, anticancer, and anti-fatigue effects through promotion of DNA, RNA, and protein synthesis^24^. In addition, ginseng contains many ingredients, such as ginsenosides, polysaccharides, polyacetylenes, fatty acids, mineral oil, peptides, and amino acids^25^. The bioactivity of ginseng is attributable to the presence of ginsenosides in the root^26^. Thus far, studies have focused on the effects of ginseng roots^26^. However, these ingredients were also distributed in other parts of ginseng, such as berries and leaves^26^. Ginseng berry was reported to have a higher ginsenoside content than roots, and its anti-hyperglycemia and anti-obesity activities were studied^27,28^. Despite its efficacy, ginseng berry has not received much attention^29^. No studies have been reported on the effects of ginseng berry extract in improving blood circulation. Therefore, the purpose of this study was to determine whether tumor necrosis factor (TNF)-□-induced endothelial cells and muscle cells would be recovered using ginseng berry extract and to investigate the effect of the extract intake on blood circulation. NO is expected to be produced in vascular endothelial cells owing to treatment with the extract, and the produced NO is expected to increase cGMP level and decrease the level of adhesion molecules in vascular muscle cells.

## METHODS

### Preparation of ginseng berry extract

Raw ginseng berries were harvested from Hongcheon, Gangwon-do, South Korea, and the seeds were separated and removed. Ginseng berries were dried and refluxed with 100% distilled water and concentrated under reduced pressure at 45˚C (the patent number 10-2015-0101209).

### Cell culture

Human umbilical vein endothelial cells (HUVECs) and smooth muscle cells (A7r5) were both purchased from ATCC (Manassas, VA, USA). HUVECs were cultured in complete microvascular endothelial cell growth medium (EGM-2, Lonza Walkersville, Inc.), in humidified 5% CO_2_ incubator at 37˚C. A7r5 cells were cultured in complete medium (DMEM, Hyclone, GE Healthcare Life Sciences, South Logan, Utah, USA), in humidified 5% CO_2_ incubator at 37˚C.

### A7r5 and HUVEC co-culture

Co-culture plates, SPLINsertTM Hanging plates were purchased from SPL (SPL Life Sciences, Pochun, Gyeonggi-do, South Korea). A7r5 cells were seeded at a density of 1 × 105 cells per well in complete DMEM, and HUVECs were seeded at a density of 5 × 104 cells per cassette in complete EGM-2. Cassettes with HUVECs were placed on each well and incubated in humidified 5% CO_2_ incubator at 37˚C.

### Cell viability

HUVECs were seeded at a density of 1 × 104 cells per well in a 96-well plate. Cells were incubated for 24 h with EGM-2, in humidified 5% CO_2_ incubator at 37˚C. After 24 h, the medium was removed and cells were washed with 1× DPBS (Hyclone). Fresh medium was added and ginseng berry extract at various concentrations (0, 15. 63, 31. 25, 62. 5, 125, 250 and 500 μg/ml) was added. TNF-α (10 ng/ml) was added after 30 min and the cells were incubated in humidified 5% CO_2_ incubator at 37˚C. A Cell counting Kit-8 (Dojindo, Rockville, MD, USA) was used to determine the viability of HUVECs. CCK-8 solution (10 μl) was added in each well and incubated for 4 h in humidified 5% CO_2_ incubator at 37˚C. The absorbance was measured at 450 nm with a plate reader (Varioskan Lux, Thermofisher).

### Total RNA extraction and cDNA synthesis

HUVECs were seeded at a density of 2 × 105 cells per well and incubated for 24 h. Culture medium without FBS was added, and ginseng berry extract at various concentrations (20, 100, and 500 μg/ml) was added. TNF-α (10 ng/ml) was added to each group (except normal cell group) and incubated for 24 h. Cells were washed with 1× DPBS and treated with easy-BLUETM (1 ml) of the Total RNA Extraction Kit (Intron Biotechnology, Sungnam, Gyeonggi-do, South Korea). Cell lysates were collected, 200 μl of chloroform was added, and centrifuged at 10,000 ×g (4˚C, 10 min). Supernatants were collected, iso-propanol (500 μl) was added, and the mixture was kept for standing for 10 min at room temperature. The mixed solution was centrifuged at 7,500 ×g (4˚C, 10 min), and the supernatants were discarded. Pellets were washed with 75% ethanol and centrifuged at 7,500 ×g (4˚C, 5 min) twice. Pellets were dissolved with distilled water and treated with DEPC. Extraction of total RNA from A7r5 cells, which were co-cultured with HUVECs, was performed following the same procedure.

cDNA synthesis was performed with Power cDNA Synthesis Kit (Intron Biotechnology), according to the manual in the kit. Total RNA (1 μg) was used to synthesize cDNA, which was used to perform reverse transcription-polymerase chain reaction.

### Intracellular nitric oxide production in HUVECs

HUVECs were seeded at a density of 2 × 104 cells per well in a 96-well black plate (SPL Life Sciences, Pochun, Gyeonggi-do, South Korea) and incubated in humidified 5% CO_2_ incubator at 37˚C. The culture medium was changed to FBS-free culture medium after 24 h, and ginseng berry extract at various concentrations (20, 100, and 500 μg/ml) was added to each well. TNF-α (10 ng/ml) was added after 30 min. Detection of intracellular NO was performed according to the kit, OxiSelectTM Intracellular Nitric Oxide (NO) Assay Kit (Cell Biolabs, San Diego, CA, USA) manual and fluorescence was measured with a fluorometric plate reader at 480 nm/530 nm.

### cGMP quantification in A7r5 cells

A colorimetric cGMP ELISA Kit (Cell Biolabs) was used to determine the cGMP level in A7r5 smooth muscle cells. A7r5 cells were co-cultured with HUVECs and the samples were treated with or without with TNF-α for 24 h in humidified 5% CO_2_ incubator at 37˚C. Cells were lysed and the cGMP level was determined in the lysates following the kit manual. At the end of the experiment, the absorbance was read at 450 nm using a plate reader.

### Reverse transcription-polymerase chain reaction

Primers for HUVECs, β-actin, eNOS, ET-1, VCAM-1, ICAM-1, e-selectin, and p-selectin mRNA specific primers were synthesized and purchased from Macrogen (Seoul, South Korea). Primers for A7r5, GAPDH, VCAM-1, ICAM-3, e-selectin, and p-selectin were also synthesized and purchased from Macrogen (Table 1). Maxime PCR PreMix Kit (i-Taq) from Intron was used to perform PCR amplification. PCR products were loaded on 1% agarose gel to perform electrophoresis.

**Table 1.** Blood circulation changes following ginseng berry extract intake

	Group	Pre	Post	*p* value
RPWV, cm/sec	GBE	1222. 73±203. 80	1208. 93±209. 75	0. 44
	PG	1213. 38±163. 32	1226. 31±201. 27	
LPWV, cm/sec	GBE	1239. 53±212. 07	1225. 13±210. 61	0. 66
	PG	1227. 85±153. 63	1227. 85±153. 63	
RABPI	GBE	1. 15±0. 08	1. 13±0. 08	0. 016
	PG	1. 06±0. 07	1. 11±0. 10	
LABPI	GBE	1. 12±0. 09	1. 14±0. 08	0. 57
	PG	1. 10±0. 11	1. 10±0. 11	

Mean ± Standard deviation (SD)

GBE; ginseng berry extract supplements group, PG; placebo supplements group, RPWV; right brachial ankle pulse wave velocity, LPWV; left brachial ankle pulse wave velocity, RABPI; right ankle brachial pressure index, LABPI; left ankle brachial pressure index

**Table 2.** Primers used for reverse transcription-PCR

Gene	Cell Line	Direction	Sequence (5’ → 3’)
β-actin	HUVEC	Sense	CCAGGTCATCACCATTGG
		Antisense	CAGAGTACTTGCGCTCAG
eNOS	HUVEC	Sense	ACGGCCTCTTTCATGAAGCA
		Antisense	GGGATCAAAAGCCTTCCGGA
ET-1	HUVEC	Sense	ACTCAGGGCTGAAGACAT
		Antisense	TGACGCTGTTTCTCATGG
VCAM-1	HUVEC	Sense	AATGGGAGCTCTGTCACT
		Antisense	CTCTGCCTTTGTTTGGGT
ICAM-1	HUVEC	Sense	CAGCTCCAGACCTTTGTC
		Antisense	CACTTCACTGTCACCTCG
e-selectin	HUVEC	Sense	GCTGTGAGGAGGGATTTG
		Antisense	GCAGAAAGTCCAGCTACC
p-selectin	HUVEC	Sense	TGGAATGATGAGCACTGC
		Antisense	TCATGAGCACGTGTTGAG
GAPDH	A7r5	Sense	GTCAAGGCTGAGAATGGG
		Antisense	GTCTTCTGAGTGGCAGTG
VCAM-1	A7r5	Sense	CCCAAACAAAGGCAGAGT
		Antisense	ATGTCTCCTGTCTTGGCT
ICAM-1	A7r5	Sense	CATCCCACAGAAGCCTTC
		Antisense	CAGGATGAGGTTCTTGCC
e-selectin	A7r5	Sense	GGAAGCTAAGAACTGGGC
		Antisense	CAGGTTGGGTCAAAGCTT
p-selectin	A7r5	Sense	TAGTGGCCATCCAGAACA
		Antisense	TTCACACTCTGGCCCATA

### Subject characteristics and ginseng berry extract administration

In this study, 30 males (over 20 years of age) were included and divided into the following groups: Ginseng berry extract group (n=15; age, 34. 73 ± 6. 97 years; height, 172. 33 ± 7. 20 cm; weight, 73. 33 ± 12. 19 kg) and placebo group (n=13; age, 35. 07 ± 10. 25 years; height, 170. 79 ± 9. 19 cm; weight, 65. 43 ± 14. 67 kg). Two subjects in the placebo group voluntarily discontinued after 1 week of initiating the administration of ginseng berry extract and were excluded from the study. Thus, 28 patients orally took ginseng berry extract for 4 weeks, and ginseng berry extract distributed to the subjects completed an average intake of 66%. Ginseng berry extract was administered in the form of hard capsules containing ginseng fruit extract powder (500 mg strength); the capsules were administered twice daily to ensure a dose of 1000 mg per day. The intake setting was based on a significant increase in serum NO based on the previous animal study result^9^.

### Evaluation of improvement of blood circulation

To evaluate the improvement of blood circulation, brachial ankle pulse wave velocity and ankle brachial pressure index were measured before and after ingestion of ginseng berry extract. All subjects randomly assigned to the ginseng berry extract group visited the laboratory before and 4 weeks after ingestion and took a rest for 10-15 minutes while they were in stable condition. During the resting period, the heart rate reached 60 bpm, and the stable heart rate was maintained for more than 2 min. All measurements were performed using a non-invasive vascular screening device (VP-1000 plus, Omron Healthcare Co., Ltd., Japan).

### Data analysis

All data were analyzed using SPSS 21. 0 statistical package, and the mean and standard deviation were calculated. The changes in NO and cGMP levels were tested by independent t-tests. In addition, independent t-test was used to determine the difference among subject characteristics and baseline blood circulation values before the administration of ginseng berry extract, and 2-way mixed ANOVA was used to test the difference between groups before and after ingestion of ginseng berry extract. The significance test level was set at *p* < 0.05.

## RESULTS

### Effects of ginseng berry extract on HUVEC viability

**Fig. 1.**
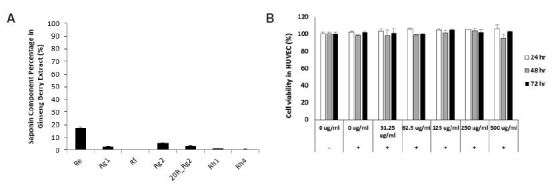
Ginseng berry extract contents and cell viability in HUVEC.Component of Ginseng Berry and Cell Viability in HUVECs. (A) Percentage of Ginsenoside Re, Rg1, Rf, Rg2, 20R_Rg2, Rh1 and Rh4 in Ginseng Berry Extract. (B) Cell Viability in HUVECs treated with Ginseng Berry extract 0, 31.25, 62.5, 125, 250 and 500 ug/ml were measured by CCK-8 kit.

No cytotoxicity or adverse effects were observed in HUVECs even after treatment with the highest concentration of the ginseng berry extract (500 μg/ml; Figure 1). Since no cytotoxicity was observed at up to 500 μg/ml, we used the extract at a concentration up to 100 μg/ml in subsequent experiments.

### Ginseng berry extract induces eNOS expression, which increases intracellular NO levels in HUVECs

**Fig. 2.**
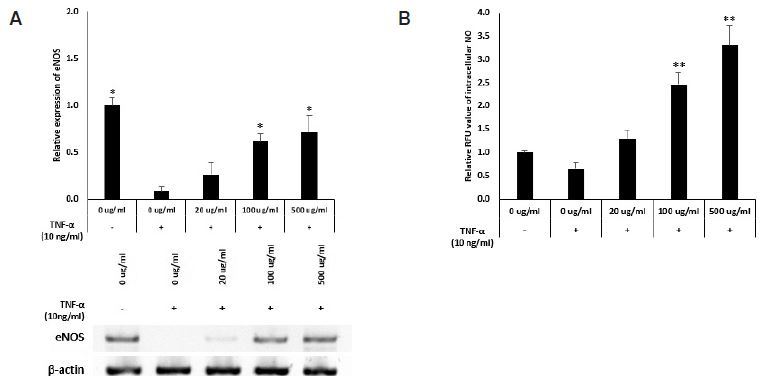
Ginseng berry Extract induces eNOS expression which leads increase of intracellular NO in HUVEC. eNOS Expression and Intacellular NO level in HUVEC. (A) eNOS exprssion level and (B) intracellular NO level in HUVECs, treated with 0, 20, 100 and 500 ug/ml. (mean ± s.d., *P<0.05, **P<0.01 as compared to TNF-a-induced Ginseng berry 0 ug/ml treated group).

TNF-α-induced cells showed downregulated eNOS expression and decreased NO production compared to normal cells. Treatment with the ginseng berry extract recovered the expression of eNOS and thereby the production NO. This result confirms that the ginseng berry extract affects the production of NO (Figure 2).

### Ginseng berry extract regulates adhesion molecules through the NO-cGMP pathway in A7r5 muscle cells

HUVECs and A7r5 cells were co-cultured. Treatment with TNF-α decreased cGMP level, which was recovered by the ginseng berry extract in a dose-dependent manner. This result indicates that the ginseng berry extract has a significant effect on the NO-cGMP pathway.

**Fig. 3.**
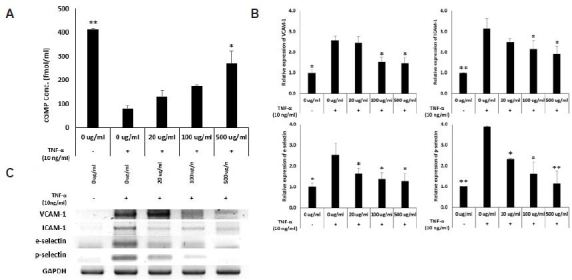
Ginseng berry Extract reduce TNF-a-induced adhesion molecules through NO-cGMP pathway in A7r5. RT-PCR results of cGMP and Adhesion Molecules Expression level by Ginseng berry extract in A7r5. (A) Reletive expression of cGMP and (B) adhesion molecules in A7r5, treated by 0, 20, 100 and 500 ug/ml Ginseng berry extract and (C) RT-PCR results, treated with 0, 20, 100 and 500 ug/ml. (mean ± s.d.,P *<0.05, **P<0.01 as compared toT NF-a-induced Ginseng berry 0 ug/ml treated group).

The expression of the adhesion molecules VCAM-1, ICAM-1, e-selectin, and p-selectin increases in endothelial cells in response to excessive diet or stress. The results show that the expression of adhesion molecules increased in TNF-α-induced A7r5 cells and was reduced in cells treated with the ginseng berry extract (Figure 3).

### Ginseng berry extract reduces TNF-α-induced adhesion molecule and ET-1 expression in HUVECs

**Fig. 4.**
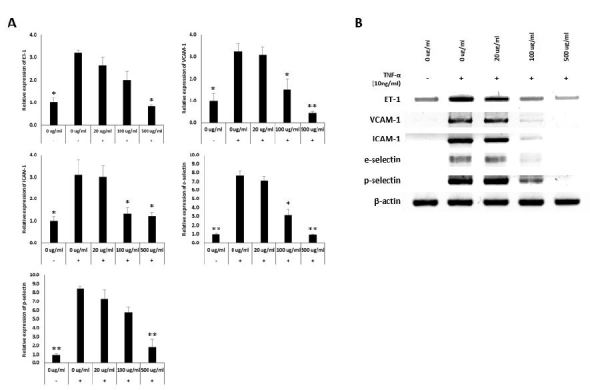
Ginseng berry Extract reduce TNF-a-induced adhesion molecules and ET-1 expression in HUVEC. RT-PCR results of Adhesion Molecules and ET-1 Expression level by Ginseng berry extract in HUVEXs. (A) Reletive expression of adhesion molecules and ET-1 in HUVEXs, treated by 0, 20, 100 and 500 ug/ml Ginseng berry extract and (B) RT-PCR results, treated with 0, 20, 100 and 500 ug/ml. (mean ± s.d., *P<0.05, **P<0.01 as compared to TNF-a-induced Ginseng berry 0 ug/ml treated group).

The increased expression of adhesion molecules and ET-1 in TNF-α-induced HUVECs was decreased by treatment with the ginseng berry extract (Figure 4).

### Blood circulation changes following the intake of ginseng berry extract

This study confirmed the relaxation of smooth muscle cells by the ginseng berry extract through in vitro experiments and clinical trials. There was no significant difference in age and physical characteristics among the participants in this study. In addition, there was no significant difference between the groups in blood circulation factors (RPWV, LPWV, RABPI, LABPI) at baseline (*p*>0. 05). There was no interaction effect among RPWV, LPWV, and LABPI in the ginseng berry extract supplement group, but RABPI decreased in the ginseng berry extract supplement group and significantly increased in the placebo group (*p*=0. 016; Table 1).

## DISCUSSION

TNF-α decreases eNOS expression and intracellular NO level and markedly increases the expression of adhesion molecules and ET-1 in endothelial cells and muscle cells, resulting in endothelium dysfunction. The results showed that these abnormalities prevented endothelium dysfunction. Many ginsenosides in ginseng berry, including ginsenoside Re, are reported to have effect on penile erection in in vivo studies. Erectile dysfunction is also associated with blood circulation and muscle contraction and relaxation^30^. The problem of blood circulation is caused by endothelial dysfunction. Endothelial dysfunction has been reported to result from a reduction in NO level, which is also referred to as impaired vasodilation^31^. The decrease in NO synthesis is due to a decrease in the expression of eNOS, a deficiency in substrate or cofactor required for eNOS activity, a change in cell signal transduction, impaired eNOS activation, and reactive oxygen species (ROS)^32-35^.

**Fig. 5.**
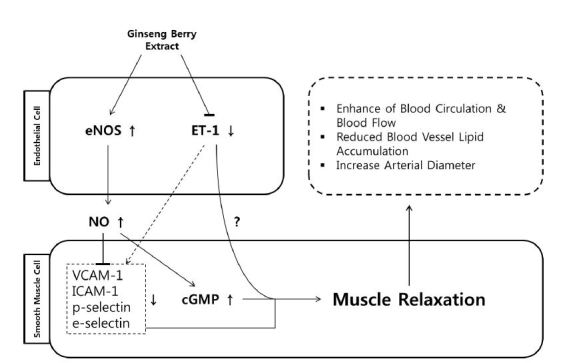
Schematic diagram of ginseng berry-blood circulation

As expected, the ginseng berry extract first increased the expression of eNOS and decreased the expression of ET-1 in endothelial cells. Increased expression of eNOS in endothelial cells eventually leads to NO production, which leads to the expression of cGMP in smooth muscle cells. Increased expression of cGMP causes smooth muscle cell relaxation and ultimately induces vasodilation. In addition, expression of VCAM-1, ICAM-1, p-selectin, and e-selectin increased by high-fat diet was reduced by ginseng berry extract, resulting in smooth muscle cell relaxation. It is known that ET-1 is strongly expressed in obese conditions in which muscle cells shrink to interfere with blood flow; however, the mechanism is not clear (Figure 5).

A decrease in blood pressure and PWV levels was observed after ingesting ginseng berry extract for a certain period of time. Increasing the diameter of the blood vessel or decreasing the thickness of the vessel wall should occur to confirm PWV reduction. In vitro experiments using vascular endothelial cells and smooth muscle cells showed that ginseng berry relaxed muscles. When the muscles relax, the thickness of the vessel wall decreases and the blood vessels expand. Based on the results of our in vitro experiments and clinical trials, we found that the ginseng berry extract helps to improve blood circulation.

## CONCLUSION

This study confirmed the effect of ginseng berry on blood circulation. Because of the difficulty in elucidating the clinical outcome, we constructed an in vitro system with two different cell lines. The change in the NO-cGMP pathway induced by ginseng berry extract was confirmed. Moreover, ginseng berry extract also decreased the expression of cell adhesion molecules causing inflammation in the blood vessel. As a result, this study suggests that the intake of ginseng berry extract improves blood circulation and therefore, ginseng berry extract intake would be helpful for people having problems with blood vessel function.
